# Indoxyl sulfate – the uremic toxin linking hemostatic system disturbances with the prevalence of cardiovascular disease in patients with chronic kidney disease

**DOI:** 10.1186/s12882-017-0457-1

**Published:** 2017-01-25

**Authors:** Tomasz W. Kamiński, Krystyna Pawlak, Małgorzata Karbowska, Michał Myśliwiec, Dariusz Pawlak

**Affiliations:** 1 Department of Pharmacodynamics, Medical University of Bialystok, Mickiewicza 2C Str., 15-222 Białystok, Poland; 2 Department of Monitored Pharmacotherapy, Medical University of Bialystok, Mickiewicza 2C Str., 15-089 Białystok, Poland; 3 Department of Nephrology and Clinical Transplantation, Medical University of Bialystok, Żurawia 14 Str., 15-540 Bialystok, Poland

**Keywords:** Indoxyl sulfate, Hemostatic disorder, Prothrombotic state, Chronic kidney disease, Monocytes activation, Uremic toxin, Tryptophan derivatives, Tissue factor, von Willebrand factor, Cardiovascular disease

## Abstract

**Background:**

During chronic kidney disease progression, kidney-specific risk factors for cardiovascular disease come into play. The present study investigated the impact of indoxyl sulfate, dietary tryptophan-derived uremic toxin, accumulated in the blood of patients with chronic kidney disease on hemostatic parameters, markers of inflammation, oxidative stress and monocyte to macrophage transition.

**Methods:**

Fifty-one CKD patients not undergoing hemodialysis were enrolled in the study. Coagulation factors, fibrinolytic parameters, adhesion molecules, endothelial dysfunction markers, oxidative stress as well as inflammation markers were examined using immune-enzymatic method. Indoxyl sulfate levels were assessed using high-performance liquid chromatography. Biochemical parameters were determined by routine laboratory techniques using an automated analyzers. All assessed parameters were compared with controls and subjected to cross-sectional statistical analysis.

**Results:**

Elevated concentrations of indoxyl sulfate, the vast majority of parameters affecting hemostasis, and markers of renal insufficiency conditions were observed. Part of hemostatic factors, namely tissue factor, von Willebrand factor, thrombomodulin, soluble urokinase-type plasminogen activator receptor, soluble intercellular adhesion molecule-1, soluble vascular cell adhesion protein were correlated with the fraction of indoxyl sulfate. A significant quantity of assessed parameters showed strong correlations with superoxide-dismutase, renal insufficiency rate, C-reactive protein, and neopterin. Levels of indoxyl sulfate were independently associated with markers of impaired endothelial function (thrombomodulin, adhesion molecules), oxidative stress (superoxide-dismutase) and monocytes activation determinant (neopterin), which indicate unconventional links between these systems and the role of indoxyl sulfate. Furthermore, parameters that correlated with the levels of indoxyl sulfate (von Willebrand factor, soluble urokinase-type plasminogen activator receptor, soluble intercellular adhesion molecule-1) were positively associated with the prevalence of cardiovascular disease in a CKD patients.

**Conclusions:**

The study demonstrated that in conditions of chronic exposure to uremic toxins, indoxyl sulfate seems to be one of the “missing links” between impaired renal function and prevalence of cardiovascular events, especially hemostatic disorders. The main functions of the action appear to be altered monocytes activation, intensified inflammatory process, and augmented oxidative stress by this uremic toxin.

## Background

Chronic kidney disease (CKD) is an established risk factor for the occurrence of hemostatic disorders and the increased prevalence of cardiovascular disease (CVD) [1, 2]. Traditional cardiovascular risk factors are insufficient to explain the high coincidence of CVD among CKD patients, suggesting the existence of “missing links” connecting cardiovascular system and kidney [3].

Clinical observations provide evidence of the coexistence of two opposite thrombotic and bleeding tendencies that are commonly observed in CKD patients. Disturbances in hemostasis during CKD are manifested in episodes of spontaneously induced prothrombotic states or severe bleeding symptoms and represent an important cause of the morbidity and mortality in patients with impaired renal function [1, 4]. The causes of hemostasis abnormalities in CKD are extremely complicated, depend on many factors and are still not fully understood despite many years of research.

Hypercoagulability is caused by abnormal activity of coagulation regulatory factors and the platelet hyper-reactivity, while excessive bleeding is the result of inadequate function of platelets, the coagulation cascade factors and/or intensified activity of the fibrinolysis [5]. The endothelial cells (ECs), cellular adhesion molecules (CAMs), the vessel wall and its extracellular matrix play some roles in the etiology of hemostatic disturbances [6]. Furthermore, patients with impaired renal function are at enhanced risk of inflammation, oxidative stress (SOX), fluid retention, further impairment of endothelial function, and anemia – conditions specific to renal disease [7, 8]. Above-mentioned components are firmly influenced by uremic toxins and metabolic compounds accumulated in CKD patients serum due to its inadequate renal clearance [9]. Altogether, it results in a mutual intensification of hemostatic disorders and progression of CKD.

Indoxyl sulfate (IS) is an aggressive uremic toxin that is markedly accumulated in the plasma of patients with CKD and its concentration in CKD patients can increase even 50-fold compared to healthy people [10]. IS is the end-stage product of dietary tryptophan metabolism and due to its high-affinity binding to albumins cannot be efficiently removed by hemodialysis. It exerts prooxidative and proinflammatory activity, triggers of the immune response, and stimulates the progression of CKD [11]. The compound exhibits its cellular toxicity in renal tubular cells, glomerular mesangial cells, and vascular smooth muscle cells (VSMCs) via numerously signaling pathways alterations [12]. Besides, IS inhibits ECs proliferation and viability, stimulates endothelium for secretion of chemokines and CAMs, and enhance expression of hemostasis-related molecules on their surface [13]. Recently, uremic toxins have been suggested as a potential “missing link” between CKD and the presence of CVD [3]. Many authors indicate a potential role of IS in the progression of vascular and hemostatic dysfunctions through the induction of SOX and progressive inflammatory process. According to Shivanna et al. [14] and Chitalia et al. [15] IS is a potential CKD-related prothrombotic uremic toxin that induces tissue factor (TF) in VSMCs and increases post-vascular interventional prothrombotic risk by TF-dependent manner. Recent study of Tang et al. indicated that IS exerts modulatory activities towards K^+^ channels leading to the development of arrhythmogenesis in CKD patients [16]. Previous studies conducted using in vitro methods and animal models provided presumptive evidence suggesting that IS may be considered as a molecule responsible for thrombotic events in CKD patients [14, 15]. Despite the existence of background information on the potential IS impact on hemostasis, currently, there are no studies discussing in a comprehensive manner this influence, especially during CKD, when the levels of IS are permanently increased. Taking this into consideration, the aim of our study was to investigate the potential associations between plasma IS levels and the parameters of: coagulation, fibrinolysis and endothelial function in CKD patients on conservative treatment. Because SOX, inflammation, and cellular immune activation are the recognized factors that may affect the hemostatic system in this population the markers of SOX and inflammatory state were also determined [17]. Moreover, we also wanted to determine, if IS-dependent hemostatic system disturbances were associated with the prevalence of CVD in this population.

## Methods

### Patients

Fifty-one predialysis patients with CKD (twenty females, thirty-one males) on conservative treatment, who were clinically stable and free of existing infections and autoimmune diseases, participated in the study. None of the patients received immunosuppressive therapy, lipid-lowering drugs, nonsteroidal anti-inflammatory agents, recombinant human erythropoietin, or antioxidants such as vitamin E, C, or allopurinol during the study. CKD was caused by glomerulonephritis (*n* = 23), diabetic nephropathy (*n* = 8), adult polycystic kidney disease (*n* = 7), pyelonephritis (*n* = 3), hypertensive nephropathy (*n* = 2), and other renal disease (*n* = 6). Twenty-three patients (45%) in the past suffered from the CVD defined as the occurrence of myocardial infarction, ischemic stroke, coronary revascularization procedures, angina pectoris, and typical ischemic changes on electrocardiogram. Antihypertensive treatment of the patients was as follows: calcium channel blockers (*n* = 27), ACE inhibitors (*n* = 25), β-receptor blockers (*n* = 23), α-receptor blockers (*n* = 4), nitrates (*n* = 3), and angiotensin-II receptor blockers (ARBs) (*n* = 2). Nine patients (17,6%) were smokers. Drugs affecting hemostasis were not administered two weeks prior the study.

Eighteen healthy volunteers matched for age and gender served as a control group. They were not taking any medications, dietary supplementation, and were on a standard diet. In the past, hypertension, CKD, diabetes mellitus, and vascular diseases was not reported. The volunteers were not taking any drugs affecting hemostasis two weeks prior the study. Table 1 shows basal characteristics of CKD patients and control group.

**Table 1:** Biochemical and clinical characteristics of control group and CKD patients

Parameter	Controls *n* = 18	CKD *n* = 51	*P* value
Sex M/F	7/11	20/31	NS
Age, years	47.4 ± 6.18	53.3 ± 15.5	NS
BMI, kg/m^2^	25.83 ± 3.45	24.2 ± 3.55	NS
eGFR, mL/min/1.73 m^2^	117.0 (105.0 – 125.0)	20.6 (5.6 - 127)	0.0001
Creatinine, mg/dL	0.88 (0.34 – 1.18)	3.32 (0.78 – 9.33)	0.0001
Urea, mg/dL	30.03 ± 6.05	118 ± 53.1	0.0001
hs-CRP, μg/ml	0.42 (0.01 – 9.25)	3.38 (0.01 - 94)	0.0079
Neopterin, nmol/L	5.93 (0.41 – 12.9)	32 (5 – 150)	0.0001
Cu/Zn SOD, ng/ml	51 (6 – 78)	62 (30 – 262)	0.0372
H_2_O_2_, μM	55.2 (2.33 – 434)	243 (60.7 – 624)	0.0012
Glucose, mmol/L	92.1 (67–114)	90 (45–186)	NS
Total cholesterol, mg/dL	192 (143–248)	199 (106–485)	NS
Triglycerides, mg/dL	67 (38–149)	152 (61–620)	0.0001
Total protein, g/dL	6.36 (6.08 – 7.1)	6 (3.2 – 7.8)	0.0261
Albumin, g/dL	4.43 (4.11 – 4.98)	3.3 (0.9 – 8.9)	0.0001
Red blood cells, ×10^3^ μL	4.55 ± 0.31	3.59 ± 0.69	0.0001
White blood cells, ×10^3^ μL	5.77 ± 1.09	6.38 ± 1.98	NS
Lymphocytes, %	33.1 ± 5.38	27 ± 9.85	0.0107
Platelets, ×10^3^ μL	206 (132.0 – 310.0)	184 (76–482)	NS
Neutrophils, %	59.19 ± 6.424	59.4 ± 13.1	NS
Hemoglobin, g/dL	14.18 ± 1.3	11.1 ± 2.31	0.0001
Hematocrit, %	42 ± 3.1	33.2 ± 6.15	0.0001
Bilirubin, mg/dL	0.34 ± 0.13	0.48 ± 0.22	0.0157
ALT, U/L	34.5 (16 – 53)	28 (10–130)	NS
Smokers, %	20.00	22.65	NS
	Pharmacotherapy (%)		
Cardiovascular Disease (CVD)		45.28	
ACE-inhibitors		49.00	
Ca^2+^ blockers		56.50	
β-blockers		41.50	
α-blockers		7.50	
Nitrates		5.50	
ARBs		4.00	

Data are shown as mean ± SD or median (range) depending on their normal or skewed distribution

*Abbreviations*: *Sex M* male, *Sex* F female, *BMI* body mass index, *eGFR* estimated glomerular filtration rate, *hs-CRP* high sensitivity C-reactive protein, *Cu/Zn SOD* superoxide dismutase 1, *H*_*2*_ *O*_*2*_ hydrogen peroxide, *ALT* alanine transaminase, *ACE-inhibitors* angiotensin-converting-enzyme inhibitor, *ARBs* angiotensin receptor blockers, *CKD* chronic kidney disease, *NS* non-significant

The study protocol was approved by the ethical guidelines of Local Ethical Committee in Bialystok and written consent was obtained from each subject.

The study was carried out in accordance with the Declaration of Helsinki.

### Blood sampling

Blood was collected from CKD patients and control group from an antecubital vein in the morning between 8 am and 9 am. Sodium citrate (3,8% in proportion 1 + 9 v/v) was used as an anticoagulant. Citrated plasma and serum samples were prepared conventionally, aliquoted, and stored at −80 °C until assayed.

### IS determination

IS was determined by liquid chromatography with fluorescence detection according to Al Za'abi et al. [18]. The chromatographic equipment was an Agilent 1200 series LC-system (Agilent Technologies, Germany) composed of G1322A degasser, G1311A quaternary pump, G1329A autosampler and G1330B thermostat for autosampler, HP1046A fluorescence detector (FLD). Deproteinated samples were prepared by adding 0.4 ml acetonitrile containing the methyl paraben (1 mg/ml) as internal standard into the 0.1 ml plasma. The samples were vortexed, kept at 4 °C for 1 min, and then centrifuged for 30 min 14000 g at 4 °C, 1 ml of the supernatant was injected into HPLC system for analysis. The prepared samples were separated on column Phenomenex PEPTIDE 3.6 mm XB-C18 4.6x250mm. The column effluent was monitored by using programmable FLD. The optimized conditions were determined by recording fluorescence spectra with a stop-flow technique. Excitation and emission wavelengths were set at 280/375 nm. The output of the detector was connected to a single instrument LC ChemStation. The mobile phase was composed of acetate buffer (pH 4.5) containing 90% of acetonitrile and it was pumped at a flow-rate of 0.8 ml/min. Chromatography was carried out at 24 °C.

### Parameters of hemostatic system

A) *Coagulation system*
  Coagulation system activation was reflected by the levels of TF and its pathway inhibitor (TFPI) in the plasma, determined by ELISA kits (IMUBIND Tissue Factor, IMUBIND Total TFPI Elisa Kit, American Diagnostica, Greenwich, CT, USA). Prothrombin fragments F_1+2_ (F_1+2_) were measured by an ELISA kit (Dade-Behring, Marburg, Germany).
B) *Fibrinolytic system*
  The plasma levels of tissue plasminogen activator (tPA), urokinase-type plasminogen activator (uPA), its soluble receptor (suPAR), PAI-1, and plasmin-antiplasmin complex (PAP) were measured by ELISA method using commercially kits (IMUBIND tPA ELISA, IMUBIND PAI-1 ELISA, IMUBIND uPA, IMUBIND suPAR from American Diagnostica, Greenwich, CT, USA; Plasmin-α-2-antiplasmin Complex from Technoclone, Austria).
C) *Endothelial function markers*
  TM and vWF-antigen plasma levels were studied using commercially available kits (Thrombomodulin ELISA Kit, American Diagnostica and Asserachrom vWF, Diagnostica Stago; respectively). Circulating forms of intercellular adhesion molecule-1 (sICAM-1) and vascular adhesion molecule-1 (sVCAM-1) were determined by commercially available ELISA kits (R&D Systems Europe, Abingdon, UK).
D) *Biomarkers of inflammation and oxidative stress*
  Plasma C-reactive protein levels (hs-CRP) were measured by high-sensitivity ELISA kits (Imuclone hs-CRP ELISA, American Diagnostica, Greenwich, USA). Plasma Cu/Zn superoxide dismutase (Cu/Zn SOD) levels were measured by ELISA kit (Bender Med Systems, Vienna, Austria). Total hydrogen peroxide (H_2_O_2_) concentrations were measured with an Oxy Stat colorimetric assay kit (Biomedica, Vienna, Austria). Neopterin concentrations were also determined by ELISA method (Demeditec Diagnostics, Kiel, Germany).

Biochemical parameters were determined by routine laboratory techniques using an automated analyzers.

### Statistical analysis

The normally distributed data were presented as mean ± 1SD, while the non-Gaussian data as median (full-range). Normality of distribution was tested using Shapiro-Wilk *W* test. The Student *t* test or nonparametric Mann–Whitney test were used to compare differences between CKD group and control group. The *χ*2 test was used for categorical variables. The correlations were analyzed using Spearman’s rank correlation analysis or quasi-Newton and Rosenbrock’s regression analysis. Multiple regression analysis was performed using a stepwise model with a forward elimination procedure to determine the combined influence of variables on particular parameters of the hemostatic system. Multiple regression analysis were performed based on previous results of Spearman’s rank correlation analysis or quasi-Newton and Rosenbrock’s regression analysis. A two-tailed *p* < 0.05 was considered statistically significant. Computations were performed using GraphPad 6 Prism (GraphPad Software; La Jolla, California, USA).

## Results

### Basal characteristics of CKD patients

CKD group did not differ from controls with regard to gender, age, body mass index, and smoking status. CKD patients compared to controls showed statistically significant (*p* < 0.0001) decreased eGFR values and increased creatinine and urea levels. We also indicated significantly elevated values of markers of SOX (Cu/Zn SOD, H_2_O_2_), monocyte activation (neopterin) and inflammation (hs-CRP) in CKD group compared to controls. The glucose levels and total cholesterol did not differ between both groups. However, we found increased values of triglycerides (*p* < 0.0001) and decreased levels of total protein and albumins (*p* = 0.026 and *p* < 0.0001; respectively). Among morphology parameters, we did not find any changes in the levels of white blood cells, platelets, and neutrophils. The CKD group showed significantly decreased counts of red blood cells, lymphocytes, hemoglobin, and hematocrit. Among CKD patients, 45% of them experienced the presence of CVD. The most frequent used medicines were ACE-inhibitors, calcium channel blockers, and β-blockers. The most rarely used drugs were nitrates and angiotensin receptor blockers. All data are showed in Table 1.

### IS levels and CKD stage

**Fig. 1:**
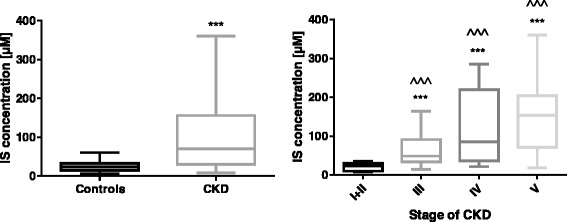
The levels of IS in control group and CKD group (left) and comparison of the levels of IS among the patients with the different stage of CKD (right). *** *p* < 0.001 controls vs CKD group; ^^^ *p* < 0.001 patients with CKD with III (*n* = 10) /IV (*n* = 9) /V (*n* = 21) stage vs I + II stage (*n* = 11). Abbreviations: IS - indoxyl sulfate; CKD - chronic kidney disease; NS - non-significant

The plasma level of IS was about three-fold higher in CKD group compared to controls (*p* < 0.0001). Levels of IS in controls did not significantly differ from IS concentrations in the earliest I + II stages of CKD, whereas the IS levels were significantly higher in stage III, IV, and V of CKD compared to controls (*p* < 0.0001). The significantly higher IS concentrations were also observed in stage III-V compared with stage I + II, (*p* < 0.0001), as presented in Fig. 1. IS level correlated with renal dysfunction markers: eGFR, creatinine (*R* = −0.685 and *r* = 0.707, *p* < 0.0001; respectively), and with urea concentration (*R* = 0.297, *p* < 0.05).

### Parameters of hemostatic system and their associations with IS concentrations, kidney function markers, SOX and inflammatory status of CKD patients

As shown in Table 2, TF concentrations in CKD group were significantly higher compared to controls (*p* < 0.0001). The marker of prothrombotic state - F1 + 2 was significantly elevated in patients in comparison to controls. There were no significant differences between TFPI levels in CKD and healthies. Furthermore, the increase of TF levels leads to increased TF/TFPI ratio with statistical significance (*p* < 0.0001). All analyzed parameters of the fibrinolysis and the markers of endothelial dysfunction were significantly higher in CKD patients than in controls.

**Table 2:** Parameters of coagulation, fibrinolysis and endothelial function markers in healthy controls and CKD patients

	Factor	Controls	CKD	*P* value
Coagulation	TF [pg/ml]	112 (10 – 316)	270 (45 – 1355)	<0.0001
	F1 + 2 [nmol/ml]	1.04 (0.66 – 2)	3.42 (0.25 – 14.2)	<0.0001
	TFPI [ng/ml]	98.4 (62.2 – 165)	78.8 (40.4 – 194)	NS
Fibrinolysis	PAP [ng/ml]	216 (31 – 541)	466 (83.9 – 1735)	0.0085
	uPA [ng/ml]	0.4 (0.1 – 1.1)	1.15 (0.6 – 5)	<0.0001
	suPAR [ng/ml]	0.07 (0.07 – 0.2)	2.42 (0.6 – 6.8)	<0.0001
	tPA [ng/ml]	5.05 (2.8 – 8.9)	7.4 (2 – 70)	0.0096
	PAI-1 [ng/ml]	24.5 (13 – 79)	55.7 (12.5 – 96.7)	0.0002
Endothelial function	vWF [ng/ml]	74.6 ± 9.59	102 ± 13.4	<0.0001
	TM [ng/ml]	2.8 ± 1.11	10.1 ± 4.63	<0.0001
	sICAM-1 [ng/ml]	226 (128 – 286)	263 (143 – 763)	0.0009
	sVCAM-1 [ng/ml]	565 (240 – 986)	912 (288 – 3426)	0.0064

Data are shown as mean ± SD or median (range) depending on their normal or skewed distribution

*Abbreviations*: *TF* tissue factor, *F1 + 2* prothrombin fragments 1 + 2, *TFPI* tissue factor pathway inhibitor, *PAP* plasmin-α2-antiplasmin, *uPA* urinary plasminogen activator, *suPAR* soluble urokinase-type plasminogen activator receptor, *tPA* tissue plasminogen activator, *PAI-1* plasminogen activator inhibitor-1, *vWF* von Willebrand Factor, *TM* thrombomodulin, *sICAM-1* soluble intercellular adhesion molecule-1, *sVCAM-1* soluble vascular cell adhesion molecule-1, *CKD* chronic kidney disease, *NS* non-significant

**Fig. 2:**
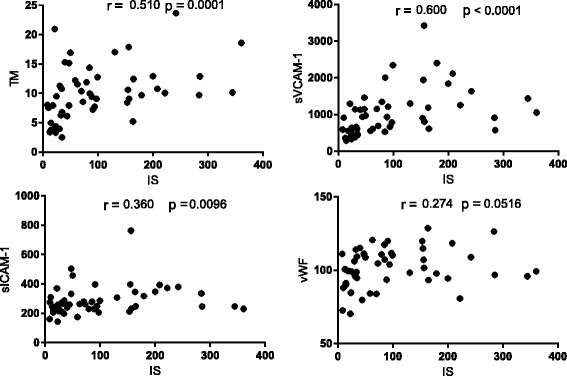
The association between plasma IS levels and the markers of endothelial function in patients with CKD. Results are shown as Spearman’s rank correlation coefficient (R) and its statistical significance (*p* values). Abbreviations: IS - indoxyl sulfate; CKD – chronic kidney disease; TM - thrombomodulin; sVCAM-1 - soluble vascular cell adhesion molecule-1; sICAM-1 - soluble intercellular adhesion molecule-1; vWF - von Willebrand Factor

**Fig. 3:**
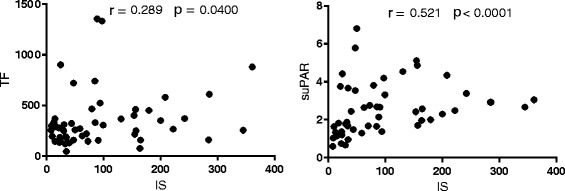
The association between plasma IS levels and TF, suPAR concentrations in patients with CKD. Results are shown as Spearman’s rank correlation coefficient (R) and its statistical significance (*p* values). Abbreviations: IS - indoxyl sulfate; CKD - chronic kidney disease; TF- tissue factor; suPAR - soluble urokinase-type plasminogen activator receptor

**Fig. 4:**
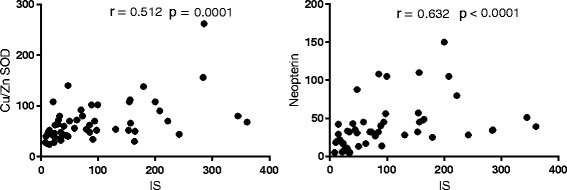
The association between plasma IS levels and the markers of oxidative stress and monocyte activation in patients with CKD. Results are shown as Spearman’s rank correlation coefficient (R) and its statistical significance (*p* values). Abbreviations: IS - indoxyl sulfate; CKD - chronic kidney disease; Cu/Zn SOD - Cu/Zn superoxide dismutase 1

We noticed the strong positive association between the concentration of IS and endothelial function markers: TM, sVCAM-1, sICAM-1 (*p* < 0.01), whereas between vWF and IS levels we observed only a weak correlation (Fig. 2). Among the analyzed parameters of coagulation and fibrinolysis, only TF and suPAR levels were significantly and positively associated with IS concentrations (Fig. 3). Moreover, we found a positive correlation between plasma levels of IS and the markers of oxidative stress: Cu/Zn SOD and H_2_O_2,_ as well as between IS and the marker of monocyte activation - neopterin (Fig. 4). Furthermore, we noticed the strong positive relationship between Cu/Zn SOD and neopterin (*R* = 0.369, *p* < 0.009), as well as between Cu/Zn SOD and hs-CRP levels (*R* = 0.304, *p* = 0.030). In contrast, there was no correlation between IS and inflammation marker - hs-CRP (*R* = 0185; NS*)*. As presented in Table 5 (Appendix), the majority of analyzed hemostatic parameters were inversely associated with kidney function marker – eGFR and some of them were positively associated with Cu/Zn SOD and hs-CRP. What is important, four of analyzed hemostatic parameters, namely TF, TM, suPAR, and sVCAM-1 were positively associated with neopterin - marker reflecting monocyte activation status.

### Variables independently associated with parameters of hemostasis, SOX and monocyte activation in patients with CKD

To examine the combined effect of factors affecting individual hemostatic parameters in CKD patients, we performed multiple regression analyses based on results of Spearman’s rank correlation analysis and quasi-Newton or Rosenbrock’s regression analysis (Table 3). TM level was the only factor independently associated with increased TF concentrations (section A). In turn, variables independently associated with plasma suPAR levels are presented in section B and variables predicting plasma endothelial dysfunction markers in patients with CKD are shown in section C. Age and F1 + 2 levels were the independent variables significantly associated with vWF concentrations in CKD, whereas PAP, IS and suPAR independently affected increased TM levels. In the case of sVCAM-1, the independent variables associated with its level in CKD group were: IS, sICAM-1, and neopterin. Moreover, sVCAM-1 and suPAR concentrations independently affected sICAM-1 levels in these patients. Because Cu/Zn SOD and neopterin were proved to be one of the factors independently affecting some parameters of the hemostatic system, the additional multiple regression analyses were performed to demonstrate the independent effect of IS on plasma levels of these markers. As is shown in section D, IS independently affected both Cu/Zn SOD and neopterin levels in CKD patients.

**Table 3:** Variables independently associated with (A) plasma TF, (B) plasma suPAR, (C) endothelial dysfunctions markers, (D) Cu/Zn SOD, and neopterin levels in patients with CKD

		Independent variable	Regression coefficient	*P* value
A	TF	TM	0.489	0.007
	Multiple R for variables in the model– 0.490, multiple R^2^ – 0.239, adjusted R^2^ – 0.211, *p* < 0.007			
B	suPAR	uPA	0.418	<0.0001
		hs-CRP	0.373	0.0002
		neopterin	0.500	0.0012
		lymphocytes	−0.249	0.0046
		TM	0.307	0.0084
		sICAM-1	0.230	0.0171
		vWF	0.157	0.0244
		Cu/Zn SOD	0.156	0.0482
	Multiple R for variables in the model – 0.985, multiple R^2^ – 0.971, adjusted R^2^ – 0.934, *p* < 0.0001			
C	vWF	age	0.371	0.0104
		F1 + 2	−0.276	0.0324
	TM*	PAP	0.497	0.0003
		IS	0.426	0.0008
		suPAR	0.399	0.0024
	sVCAM-1^	IS	0.562	0.0007
		sICAM-1	0.557	0.0008
		neopterin	0.359	0.0140
	sICAM-1#	sVCAM-1	0.547	<0.0001
		suPAR	0.357	0.0101
	Multiple R for variables in the model – 0.633 (*0.863; ^0.960; #0.785), multiple R^2^ – 0.400 (*0.745; ^0.922; #0.617), adjusted R^2^ – 0.349 (*0.713; ^0.873; #0.574), all *p* < 0.0001			
D	Cu/Zn SOD	IS	0.411	0.0031
		TFPI	0.405	0.0071
		sICAM-1	0.366	0.0093
		F1 + 2	0.250	0.0442
	neopterin*	sVCAM-1	0.659	0.0010
		TM	0.598	0.0062
		TF	0.496	0.0100
		IS	0.456	0.0119
		Hemoglobin	−0.324	0.0471
	Multiple R for variables in the model – 0.655 (*0.926), multiple R^2^ – 0.429 (*0.857), adjusted R^2^ – 0.336 (*0.786), both *p* < 0.0001			

*Abbreviations*: *TF* tissue factor, *TM* thrombomodulin, *suPAR* soluble urokinase-type plasminogen activator receptor, *uPA* urinary plasminogen activator, *hs-CRP* high sensitivity C-reactive protein, *sICAM-1* soluble intercellular adhesion molecule-1, *vWF* von Willebrand Factor, *Cu/Zn SOD* Cu/Zn superoxide dismutase 1, *F1 + 2* prothrombin fragments 1 + 2, *PAP* plasmin-α2-antiplasmin, *IS* indoxyl sulfate, *sVCAM-1* soluble vascular cell adhesion molecule-1, *TFPI* tissue factor pathway inhibitor; CKD – chronic kidney disease

### The associations between hemostatic parameters and the prevalence of CVD in CKD patients

As shown in Table 4, the strong interrelationships existed between analyzed hemostatic parameters in the plasma of CKD patients. Furthermore, we observed the associations between them and the occurrence of CVD. Among all these factors, levels of sICAM-1 (*p* < 0.01), vWF and suPAR (*p* < 0.05) were positively associated with the prevalence of CVD in this population. There was no direct relationship between the presence of CVD and IS levels (*χ* ^2^ = 0.072, NS), but the presence of CVD correlated with Cu/Zn SOD concentrations (*χ* ^2^ = 4.039, *p* = 0.044).

**Table 4:** The relationships between analyzed hemostatic parameters and cardiovascular disease (CVD) prevalence in patients with chronic kidney disease (CKD)

	vWF	TM	TF	TFPI	suPAR	uPA	tPA	sICAM
TF	−0.009	**0.415**		0.165	**0.283**	0.207	0.048	−0.039
	NS	**0.002**		NS	**0.044**	NS	NS	NS
F1 + 2	**−0.316**	0.077	−0.007	−0.013	0.069	0.101	**−0.287**	0.068
	**0.023**	NS	NS	NS	NS	NS	**0.041**	NS
TFPI	−0.165	**0.556**	0.165		**0.417**	0.210	0.067	0.104
	NS	**0.0001**	NS		**0.002**	NS	NS	NS
PAP	**0.324**	**0.302**	0.063	**0.342**	**0.346**	0.176	0.192	**0.317**
	**0.020**	**0.031**	NS	**0.014**	**0.013**	NS	NS	**0.023**
uPA	0.241	**0.493**	0.207	0.210	**0.605**		0.200	**0.372**
	NS	**0.0002**	NS	NS	**0.0001**		NS	**0.007**
suPAR	**0.479**	**0.639**	**0.283**	**0.417**		**0.605**	0.258	**0.525**
	**0.0004**	**0.0001**	**0.044**	**0.002**		**0.0001**	NS	**0.0001**
tPA	**0.307**	0.146	0.048	0.067	0.258	0.200		0.055
	**0.028**	NS	NS	NS	NS	NS		NS
PAI-1	**0.319**	−0.184	−0.173	−0.009	0.028	0.113	**0.363**	0.039
	**0.022**	NS	NS	NS	NS	NS	**0.009**	NS
vWF		0.194	−0.008	0.165	**0.479**	0.242	**0.307**	**0.277**
		NS	NS	NS	**0.0004**	NS	**0.028**	**0.049**
TM	0.194		**0.415**	**0.556**	**0.639**	**0.493**	0.146	**0.331**
	NS		**0.002**	**0.0001**	**0.0001**	**0.0002**	NS	**0.017**
sICAM-1	**0.277**	**0.331**	−0.039	0.104	**0.525**	**0.372**	0.055	
	**0.049**	**0.017**	NS	NS	**0.0001**	**0.007**	NS	
sVCAM-1	**0.288**	**0.598**	**0.282**	**0.298**	**0.656**	**0.407**	0.057	**0.654**
	**0.040**	**0.0001**	**0.044**	**0.033**	**0.0001**	**0.003**	NS	**<0.0001**
CVD	**6.400**	2.865	1.527	1.191	**5.802**	0.020	0.554	**8.881**
	**0.011**	NS	NS	NS	**0.016**	NS	NS	**0.003**

Results are shown as Spearman’s rank correlation coefficients (r) or bivariate logistic (*χ* ^2^) regression coefficient

*Abbreviations*: *TF* tissue factor, *F1 + 2* prothrombin fragments 1 + 2, *TFPI* tissue factor pathway inhibitor, *PAP* plasmin-α2-antiplasmin, *uPA* urinary plasminogen activator, *suPAR* soluble urokinase-type plasminogen activator receptor, *tPA* tissue plasminogen activator, *PAI-1* plasminogen activator inhibitor-1, *vWF* von Willebrand Factor, *TM* thrombomodulin, *sICAM-1* soluble intercellular adhesion molecule-1, *sVCAM-1* soluble vascular cell adhesion molecule-1, *CVD* cardiovascular disease, *NS* non-significant

## Discussion

The aim of our study was to examine the precise impact of IS on the hemostatic system in the aspect of the prevalence of cardiovascular incidents. Existing basis lead us to consideration that IS may be one of the “missing links” between CVD and CKD that are closely interrelated and reinforce each other [19–23]. The results of our study demonstrated for the first time that: (1) the increased plasma IS concentrations were associated with disturbances of hemostatic system, increased oxidative stress and monocyte activation in patients with CKD; (2) IS levels, oxidative stress, and monocyte activation were independently associated with part of the evaluated parameters of hemostasis; (3) accumulation of IS in the plasma of CKD patients may participate in the risk of CVD prevalence through the mechanism associated with disturbances of hemostatic system.

Our results are in line with other observations proving that IS levels are strongly correlated with stages of CKD [23]. We also found a strong positive association of IS with Cu/Zn SOD – a recognized marker of oxidative stress in CKD population [24] as well as a positive relationship between IS and neopterin, which is synthesized by monocytes and macrophages in response to interferon-*γ* produced by activated T cells [25], what is more, we demonstrated the strong and independent effect of IS on these biomarkers, which indicated the direct role of IS in the generation of SOX and monocyte activation. Moreover, another uremic toxin – p-cresol but not p-cresol sulfate (PCS) stimulates monocyte chemoattractant protein-1 (MCP-1) expression via NF-kappa B (NF-κB) p65 in VSMC [26]. Previously, IS was shown to induce SOX in cell cultures [27]. Ito et al. [28] used a nephrectomized mouse model to demonstrate that oral administration of IS activates numbers of proinflammatory functions and reactive oxygen species (ROS) production in monocytes. However, the present study is the first research demonstrating the direct effect of IS on monocyte activation in CKD patients.

In accordance with our previous [29, 30] and other findings [31], we demonstrated the abnormalities of the hemostatic system in CKD. The maintenance of hemostasis, which is a complex mechanism, depends on many factors. Both components of the hemostatic system: coagulation and fibrinolysis, are affected by impaired endothelial function, inflammation, immune response, oxidative stress and effects of accumulated toxins [32]. In the current study, the activation of coagulation cascade was reflected by increased TF level, TF/TFPI ratio, and an increment of prothrombin fragments F1 + 2 – an indicator of in vivo thrombin generation [33]. Simultaneously, the activation of fibrinolysis was observed in these patients. Among the analyzed parameters of coagulation and fibrinolysis, IS was positively associated with TF and suPAR values. However, the results of the multivariate analysis revealed that TM was the only independent factor affecting increased TF levels in CKD patients. In contrast, suPAR concentrations were independently affected by different factors, precisely by uPA, TM, inflammatory status, monocyte activation and oxidative stress (Table 3, section B). Interestingly, TF, suPAR, and TM levels were associated with neopterin and oxidative status, and the strong relationships existed between these molecules, as shown in Table 4. Although ECs are regarded as the main source of circulating TF, suPAR and TM [34], the obtained results suggest that above-mentioned molecules may partially come from the activated monocytes [35, 36]. This hypothesis is supported by observation that plasma levels of vWF, which is a reliable marker of ECs activation, did not correlate with TF or TM concentrations (Table 4). Thus, the significant elevation of circulating TF and TM, combined with their strong relationship with the monocyte activation marker – neopterin, makes activated monocytes probable (apart from endothelium) source of these molecules in CKD patients. Because in the current study IS strongly and independently affects the markers of monocyte activation and oxidative stress, we hypothesized that this uremic toxin can be partially responsible for activation of ECs and monocytes, which in turn can lead to the increased release of TF and TM. This hypothesis is supported by previous observation of Gondouin et al. [37] showing that IS, through a ROS-mediated mechanism, induces TF expression in monocytes and ECs. In the case of suPAR, which strongly correlated with vWF, neopterin and the markers of oxidative stress and inflammation, it seems that the different types of activated cells release this molecule in CKD patients [38].

Endothelial dysfunction is frequently observed in uremic conditions [32]. The present study confirmed that the markers of endothelial dysfunction like vWF, TM, sICAM-1, and sVCAM-1 were markedly elevated in the plasma of CKD group compared to controls. The strong positive associations existed between IS, as well as Cu/Zn SOD and the majority of the endothelial markers, whereas between IS and vWF only tendency to positive correlation was observed. Interestingly, all endothelial markers were affected by inflammation (Table 5 - Appendix). These results indicate that inflammatory state may be mainly responsible for endothelial dysfunction in CKD patients, and this is in line with previous observations [27]. The adhesion of circulating monocytes to endothelium is mediated by cell adhesion molecules, such as ICAM-1 and VCAM-1, which are upregulated on the ECs surface. Furthermore, ROS serves as common intracellular messengers for redox-sensitive pathways, playing a role in the expansion of vascular disease [39]. ROS can induce endothelial injury through activation of transcription factor – NF-kB, a key redox-sensitive regulator of chemokines, cytokines, and CAMs [40]. Moreover, the mechanism linking ROS with vascular inflammation has already been documented [41]. In the condition of the present study, sVCAM-1 was independently affected by plasma IS, neopterin, and sICAM-1 levels. In addition, we noticed the strong relationship between this adhesion molecule and the markers of SOX and inflammation. Although plasma sICAM-1 was independently associated with sVCAM-1 and suPAR, its levels were also related to oxidative stress and inflammatory state. What is more, there was a positive relationship between the markers of oxidative stress and inflammation in our CKD patients. On the basis of above results, we proposed the hypothesis that IS could provoke ROS production and CAMs expression, leading to monocyte-endothelial cell interaction, the initiation of vascular inflammation and endothelial dysfunction. This hypothesis is supported by the study of Ito et al. [28], who demonstrated IS-dependent ROS production in a monocytic cell line, and enhanced adhesion of these cells to vascular endothelium in vitro [28]. They also showed that IS reduction, by the administration of IS absorbent – AST-120, significantly decreased ROS level in monocytes of nephrectomized mice. Besides, Stinghen et al. [42] demonstrated with in vitro and in vivo models that exposure of the endothelium to uremic plasma results in the increase of sVCAM-1 expression, which indicated a link between vascular activation, systemic inflammation, and uremic toxicity. In addition, Tumur et al. [43] showed that IS upregulated the expression of ICAM-1 by ROS-induced activation of NF- κB in vascular ECs, and through this mechanism may play an important role in the development of CVD.

IS is one of the most investigated uremic toxins on account of its negative impact on the cardiovascular system. Clinical studies demonstrated that serum IS level is a predictor of overall and cardiovascular mortality [22, 23, 44]. On the other hand, Lin et al. proved that elevated levels of PCS and IS are associated with increased mortality in patients with CKD, while PCS, but not IS, is associated with an increased risk of cardiovascular events [45]. Although we could not establish the direct correlation between plasma levels of IS and CVD prevalence, we found an association between CVD and some hemostatic factors, such as vWF, suPAR and sICAM-1. These factors were not only associated with CVD prevalence, but also the strong interrelationships existed between them (Table 4). This fact indicates that multiple dysfunctions of the vascular cells were present among patients with CKD [46]. vWF is released into circulation by activated ECs and mediates platelet adhesion to injured endothelium – the first step in thrombus formation [47]. It is established that vWF has independent prognostic value for all-cause mortality and CV events in peritoneal dialyzed and hemodialyzed patients [48]. In accordance with these data, our previous study [30] demonstrated that vWF was independently associated with an early indicator of systemic atherosclerosis – intima-media thickness in CKD patients. An elevated suPAR level is thought to reflect activation of the inflammatory and immune systems, and it predicts cancer, CVD, diabetes and mortality in the general population [49]. The impact of suPAR on the cardiovascular system in CKD is relatively unknown. Previously, we showed that uPA/suPAR system was associated with hyperfibrinolysis, oxidative status and CVD prevalence in pre-dialysis and hemodialysis CKD patients [29, 50]. The study of Meijers et al. [31] confirmed that the higher suPAR level was directly associated with both overall mortality and cardiovascular events in the uremic population. sICAM-1 and sVCAM-1 trigger leukocyte adhesion and migration into the subendothelial space, initiating the formation of atherosclerotic lesions [39]. Data of Stenvinkel et al. [51] suggest that sICAM-1 is an independent predictor of mortality in pre-dialysis patients, who are malnourished, inflamed, and have signs of CVD.

Although it is well established that both oxidative stress, as well as monocyte activation, were associated with CVD development in uremia [52], we showed for the first time in clinical conditions that IS can be a factor linking these abnormalities with the prevalence of CVD by a common mechanism associated with the disorders of hemostatic system.

This study is limited due to its cross-sectional design that makes us unable to establish precise mechanisms underlying observed associations in view of various factors related to processes discussed in this work and characteristic for CKD. Due to relatively small numbers of patients, our results require further prospective cohort studies. Furthermore, our study did not focus on the effect of the neutralization of IS toxicity mechanisms by counteragents.

## Conclusions

In conclusion, this study demonstrated for the first time the involvement of IS in the disturbances of the hemostatic system by the mechanism associated with oxidative stress and monocytes activation, which can result in the development of CVD in CKD patients on conservative treatment. Previously, we observed the impact of kynurenines, the other components of tryptophan metabolism pathway, on disturbances of hemostasis and CVD prevalence in CKD patients [24, 29, 31]. In this context, the current study extends the knowledge concerning the impact of tryptophan metabolites on a risk of cardiovascular complications in uremia. The clinical relevance of this work may reside on the novel characterization of CKD patients’ populations at increased risk of cardiovascular events, which are dependent from hemostatic disorders.

## Appendix

**Table 5:** The Spearman’s correlation between analyzed hemostatic parameters and the markers of monocyte activation (neopterin), oxidative stress (Cu/Zn SOD), inflammation (hs-CRP), and renal function (eGFR)

	Factor	Neopterin (R /*P* value)	Cu/Zn SOD (R/*P* value)	Hs-CRP (R /*P* value)	eGFR, (R /*P* value)
Coagulation	TF	**0.338**	**0.283**	0.073	**- 0.392**
		**0.015**	**0.044**	NS	**0.004**
	F1 + 2	0.019	**0.287**	0.102	- 0.140
		NS	**0.041**	NS	NS
	TFPI	0.179	**0.325**	**0.325**	**- 0.402**
		NS	**0.020**	**0.020**	**0.003**
Fibrinolysis	PAP	0.046	0.006	**0.304**	−0.027
		NS	NS	**0.030**	NS
	uPA	0.179	**0.287**	0.247	**- 0.329**
		NS	**0.040**	NS	**0.018**
	suPAR	**0.314**	**0.427**	**0.562**	**- 0.529**
		**0.024**	**0.002**	**<0.0001**	**<0.0001**
	tPA	−0.181	- 0.183	0.114	0.121
		NS	NS	NS	NS
	PAI-1	**−0.297**	0.004	0.096	**0.281**
		**0.034**	NS	NS	**0.046**
Endothelial function	vWF	0.076	0.165	**0.330**	- 0.172
		NS	NS	**0.018**	NS
	TM	**0.393**	**0.451**	**0.300**	**- 0.707**
		**0.004**	**0.0008**	**0.033**	**<0.0001**
	sICAM-1	0.158	**0.367**	**0.496**	- 0.232
		NS	**0.008**	**0.0002**	NS
	sVCAM-1	**0.527**	**0.448**	**0.467**	**- 0.569**
		**<0.0001**	**0.0007**	**0.0006**	**<0.0001**

Results are shown as Spearman’s rank correlation coefficients (R) and its statistical significance (*P* values)

*Abbreviations*: *TF* tissue factor, *F1 + 2* prothrombin fragments 1 + 2, *TFPI* tissue factor pathway inhibitor, *PAP* plasmin-α2-antiplasmin, *uPA* urinary plasminogen activator, *suPAR* soluble urokinase-type plasminogen activator receptor, *tPA* tissue plasminogen activator, *PAI-1* plasminogen activator inhibitor-1, *vWF* von Willebrand Factor, *TM* thrombomodulin, *sICAM-1* soluble intercellular adhesion molecule-1, *sVCAM-1* soluble vascular cell adhesion molecule-1, *Cu/Zn SOD* Cu/Zn superoxide dismutase 1, *hs-CRP* high sensitivity C-reactive protein, *eGFR* estimated glomerular filtration rate, *NS* non-significant

## Abbreviations

ARBs: Angiotensin II receptor blockers
CKD: Chronic kidney disease
Cu/Zn SOD: Cu/Zn superoxide dismutase
CVD: Cardiovascular disease
ECs: Endothelial cells
F1 + 2: Prothrombin fragments 1 + 2
FLD: Fluorescence detector
H2O2: Hydrogen peroxide
hs-CRP: C-reactive protein levels
IS: Indoxyl sulfate
MCP-1: Monocyte chemoattractant protein-1
NF-kB: Nuclear factor kappa B
PAI-1: Plasminogen activator inhibitor 1
PAP: Plasmin-antiplasmin complex
PCS: p-cresol sulfate
ROS: Reactive oxygen species
sCAMs: Soluble cellular adhesion molecules
sICAM-1: Intracellular adhesion molecule-1
SOX: Oxidative stress
suPAR: Soluble urokinase-type plasminogen activator receptor
sVCAM-1: Soluble vascular adhesion molecule-1
TF: Tissue factor
TFPI: Tissue factor pathway inhibitor
TM: Thrombomodulin
tPA: Tissue plasminogen activator
uPA: Urokinase-type plasminogen activator
VSMCs: Vascular smooth muscle cells
vWF: von Willebrand Factor

## References

[1] Lutz J, Menke J, Sollinger D, Schinzel H, Thürmel K. Haemostasis in chronic kidney disease. Nephrol Dial Transplant. 2014. doi:10.1093/ndt/gft209. 10.1093/ndt/gft209 24132242

[2] Danaei G, Lu Y, Singh GM, Carnahan E, Stevens GA, Cowan MJ, et al. Global burden of metabolic risk factors for chronic diseases collaboration: cardiovascular disease, chronic kidney disease, and diabetes mortality burden of cardiometabolic risk factors from 1980 to 2010: a comparative risk assessment. Lancet Diab Endocrinol. 2014. doi:10.1016/S2213-8587(14)70102-0. 10.1016/S2213-8587(14)70102-0 PMC4572741 24842598

[3] Lekawanvijit S, Krum H. Cardiorenal syndrome: acute kidney injury secondary to cardiovascular disease and role of protein-bound uraemic toxins. J Physiol. 2014. doi:10.1113/jphysiol.2014.273078. 10.1113/jphysiol.2014.273078 PMC4198008 24907309

[4] Thomas R, Kanso A, Sedor JR. Chronic kidney disease and its complications. Prim Care. 2008. doi:10.1016/j.pop.2008.01.008. 10.1016/j.pop.2008.01.008 PMC2474786 18486718

[5] Palta S, Saroa R, Palta A. Overview of the coagulation system. Indian J Anaesth. 2014. doi:10.4103/0019-5049.144643. 10.4103/0019-5049.144643 PMC4260295 25535411

[6] Chiu JJ, Chien S. Effects of disturbed flow on vascular endothelium: pathophysiological basis and clinical perspectives. Physiol Rev. 2011. doi:10.1152/physrev.00047.2009. 10.1152/physrev.00047.2009 PMC3844671 21248169

[7] Colombo PC, Ganda A, Lin J, Onat D, Harxhi A, Iyasere JE, et al. Inflammatory activation: cardiac, renal, and cardio-renal interactions in patients with the cardiorenal syndrome. Heart Fail Rev. 2012. doi:10.1007/s10741-011-9261-3. 10.1007/s10741-011-9261-3 PMC3876739 21688186

[8] Basu G, Mohapatra A. Interactions between thyroid disorders and kidney disease. Indian J Endocrinol Metab. 2012. doi:10.4103/2230-8210.93737. 10.4103/2230-8210.93737 PMC3313737 22470856

[9] Mutsaers HA, Engelke UF, Wilmer MJ, Wetzels JF, Wevers RA, van den Heuvel LP, et al. Optimized metabolomic approach to identify uremic solutes in plasma of stage 3–4 chronic kidney disease patients. PLoS One. 2013. doi:10.1371/journal.pone.0071199. 10.1371/journal.pone.0071199 PMC3732267 23936492

[10] Niwa T (2010). Uremic toxicity of indoxyl sulfate. Nagoya J Med Sci.

[11] Barisione C, Ghigliotti G, Canepa M, Balbi M, Brunelli C, Ameri P (2015). Indoxyl sulfate: a candidate target for the prevention and treatment of cardiovascular disease in chronic kidney disease. Curr Drug Targets.

[12] Ng HY, Yisireyili M, Saito S, Lee CT, Adelibieke Y, Nishijima F, et al. Indoxyl sulfate downregulates expression of Mas receptor via OAT3/AhR/Stat3 pathway in proximal tubular cells. PLoS One. 2014. doi:10.1371/journal.pone.0091517. 10.1371/journal.pone.0091517 PMC3948887 24614509

[13] Barisione C, Garibaldi S, Furfaro AL, Nitti M, Palmieri D, Passalacqua M, et al. Moderate Increase of Indoxyl Sulfate Promotes Monocyte Transition into Profibrotic Macrophages. PLoS One. 2016. doi:10.1371/journal.pone.0149276. 10.1371/journal.pone.0149276 PMC4771744 26925780

[14] Shivanna S, Kolandaivelu K, Shashar M, Belghasim M, Al-Rabadi L, Balcells M, et al. The Aryl Hydrocarbon Receptor is a Critical Regulator of Tissue Factor Stability and an Antithrombotic Target in Uremia. J Am Soc Nephrol. 2016. doi:10.1681/ASN.2014121241. 10.1681/ASN.2014121241 PMC4696580 26019318

[15] Chitalia VC, Shivanna S, Martorell J, Balcells M, Bosch I, Kolandaivelu K, Edelman ER, et al. Uremic serum and solutes increase post-vascular interventional thrombotic risk through altered stability of smooth muscle cell tissue factor. Circulation. 2013. doi:10.1161/CIRCULATIONAHA.112.118174. 10.1161/CIRCULATIONAHA.112.118174 PMC4407990 23269489

[16] Tang WH, Wang CP, Chung FM, Huang LL, Yu TH, Hung WC, et al. Uremic retention solute indoxyl sulfate level is associated with prolonged QTc interval in early CKD patients. PLoS One. 2015. doi:10.1371/journal.pone.0119545. 10.1371/journal.pone.0119545 PMC4403985 25893644

[17] Wang Q, Liu D, Song P, Zou MH. Deregulated tryptophan-kynurenine pathway is linked to inflammation, oxidative stress, and immune activation pathway in cardiovascular diseases. Front Biosci. (Landmark Ed.). 2015;20:1116–43. 10.2741/4363 PMC4911177 25961549

[18] Al Za'abi M, Ali B, Al Toubi M. HPLC-fluorescence method for measurement of the uremic toxin indoxyl sulfate in plasma. J Chromatogr Sci. 2013. doi:10.1093/chromsci/bms103. 10.1093/chromsci/bms103 22718744

[19] Liu M, Li XC, Lu L, Cao Y, Sun RR, Chen S (2014). Cardiovascular disease and its relationship with chronic kidney disease. Eur Rev Med Pharmacol Sci.

[20] Zahran M, Nasr FM, Metwaly AA, El-Sheikh N, Khalil NS, Harba T. The role of hemostatic factors in atherosclerosis in patients with chronic renal disease. Electron Physician. 2015. doi:10.14661/1270. 10.14661/1270 PMC4590563 26435827

[21] Christiansen CF, Schmidt M, Lamberg AL, Horváth-Puhó E, Baron JA, Jespersen B, et al. Kidney disease and risk of venous thromboembolism: a nationwide population-based case–control study. J Thromb Haemost. 2014. doi:10.1111/jth.12652. 10.1111/jth.12652 25040558

[22] Lekawanvijit S, Kompa AR, Wang BH, Kelly DJ, Krum H. Cardiorenal syndrome: the emerging role of protein-bound uremic toxins. Circ Res. 2012. doi:10.1161/CIRCRESAHA.112.278457. 10.1161/CIRCRESAHA.112.278457 23139286

[23] Barreto FC, Barreto DV, Liabeuf S, Meert N, Glorieux G, Temmar M, et al. European Uremic Toxin Work Group (EUTox).: Serum indoxyl sulfate is associated with vascular disease and mortality in chronic kidney disease patients. Clin J Am Soc Nephrol. 2009. doi:10.2215/CJN.03980609. 10.2215/CJN.03980609 PMC2758258 19696217

[24] Pawlak K, Pawlak D, Mysliwiec M. Cu/Zn superoxide dismutase plasma levels as a new useful clinical biomarker of oxidative stress in patients with end-stage renal disease. Clin Biochem. 2005. doi:10.1016/j.clinbiochem.2005.02.009. 10.1016/j.clinbiochem.2005.02.009 15963971

[25] Huber C, JR Batchelor, Fuchs D. Immune response associated production of neopterin. Release from macrophages primarily under control of interferon-gamma. J Exp Med. 1984. doi:10.1084/jem.160.1.310 10.1084/jem.160.1.310 PMC2187425 6429267

[26] Maciel RA, Rempel LC, Bosquetti B, Finco AB, Pecoits-Filho R, Souza WM, et al. p-cresol but not p-cresyl sulfate stimulate MCP-1 production via NF-κB p65 in human vascular smooth muscle cells. J Bras Nefrol. 2016. doi:10.5935/0101-2800.20160024. 10.5935/0101-2800.20160024 27438970

[27] Masai N, Tatebe J, Yoshino G, Morita T. Indoxyl sulfate stimulates monocyte chemoattractant protein-1 expression in human umbilical vein endothelial cells by inducing oxidative stress through activation of the NADPH oxidase-nuclear factor-κB pathway. Circ J. 2010. doi:10.1253/circj.CJ-10-0117. 10.1253/circj.cj-10-0117 20818133

[28] Ito S, Higuchi Y, Yagi Y, Nishijima F, Yamato H, Ishii H. Reduction of indoxyl sulfate by AST-120 attenuates monocyte inflammation related to chronic kidney disease. J Leukoc Biol. 2013. doi:10.1189/jlb.0112023. 10.1189/jlb.0112023 23362306

[29] Pawlak K, Pawlak D, Mysliwiec M. Tissue factor and urokinase-type plasminogen activator system are related to the presence of cardiovascular disease in hemodialysis patients. Thromb Res. 2007. doi:10.1016/j.thromres.2007.01.011. 10.1016/j.thromres.2007.01.011 17331567

[30] Pawlak K, Buraczewska-Buczko A, Mysliwiec M, Pawlak D. Hyperfibrinolysis, uPA/suPAR system, kynurenines, and the prevalence of cardiovascular disease in patients with chronic renal failure on conservative treatment. Am J Med Sci. 2010. doi:10.1097/MAJ.0b013e3181b922a4. 10.1097/MAJ.0b013e3181b922a4 19926968

[31] Meijers B, Poesen R, Claes K, Dietrich R, Bammens B, Sprangers B, et al. Soluble urokinase receptor is a biomarker of cardiovascular disease in chronic kidney disease. Kidney Int. 2015. doi:10.1038/ki.2014.197. 10.1038/ki.2014.197 24897037

[32] Hadi HA, Carr CS, Al SJ (2005). Endothelial dysfunction: cardiovascular risk factors, therapy, and outcome. Vasc Health Risk Manag.

[33] Rosenberg RD, Bauer KA (1993). Prothrombin activation fragment assay. Clin Chem.

[34] Chavakis T, Willuweit AK, Lupu F, Preissner KT, Kanse SM (2001). Release of soluble urokinase receptor from vascular cells. Thromb Haemost.

[35] Lindmark T, Chen S. IL-10 inhibits LPS-induced human monocyte tissue factor expression in whole blood. Brit. J Haematol. 1998. doi:10.1046/j.1365-2141.1998.00808.x. 10.1046/j.1365-2141.1998.00808.x 9695979

[36] Fevang B, Eugen-Olsen J, Yndestad A, Brosstad F, Beiske K, Aukrust P, et al. Enhanced levels of urokinase plasminogen activator and its soluble receptor in common variable immunodeficiency. Clin Immunol. 2009. doi:10.1016/j.clim.2009.01.007. 10.1016/j.clim.2009.01.007 19232508

[37] Gondouin B, Cerini C, Dou L, Sallée M, Duval-Sabatier A, Pletinck A, et al. Indolic uremic solutes increase tissue factor production in endothelial cells by the aryl hydrocarbon receptor pathway. Kidney Int. 2013. doi:10.1038/ki.2013.133. 10.1038/ki.2013.133 23636172

[38] Lijnen HR, Collen D (1995). Mechanisms of physiological fibrinolysis. Baillieres Clin Haematol (Bailliera Tindall, London, UK).

[39] Griendling KK, Sorescu D, Lassègue B, Ushio-Fukai M. Modulation of protein kinase activity and gene expression by reactive oxygen species and their role in vascular physiology and pathophysiology. Arterioscler Thromb Vasc Biol. 2000. doi:10.1161/01.ATV.20.10.2175. 10.1161/01.atv.20.10.2175 11031201

[40] Chen JW, Lin FY, Chen YH, Wu TC, Chen YL, Lin SJ. Carvedilol inhibits tumor necrosis factor-alpha-induced endothelial transcription factor activation, adhesion molecule expression, and adhesiveness to human mononuclear cells. Arterioscler Thromb Vasc. 2014. doi:10.1161/01.ATV.0000145016.69181.fa. 10.1161/01.ATV.0000145016.69181.fa 15374848

[41] Kim SR, Bae YH, Bae SK, Choi KS, Yoon KH, Koo TH, et al. Visfatin enhances ICAM-1 and VCAM-1 expression through ROS-dependent NF-kappaB activation in endothelial cells. Biochim Biophys Acta. 2008. doi:10.1016/j.bbamcr.2008.01.004. 10.1016/j.bbamcr.2008.01.004 18241674

[42] Stinghen AE, Gonçalves SM, Martines EG, Nakao LS, Riella MC, Aita CA, et al. Increased plasma and endothelial cell expression of chemokines and adhesion molecules in chronic kidney disease. Nephron Clin Pract. 2009. doi:10.1159/000191205. 10.1159/000191205 19147993

[43] Tumur Z, Shimizu H, Enomoto A, Miyazaki H, Niwa T. Indoxyl sulfate upregulates expression of ICAM-1 and MCP-1 by oxidative stress-induced NF-kappaB activation. Am J Nephrol. 2010. doi:10.1159/000299798. 10.1159/000299798 20389059

[44] Lin CJ, Pan CF, Liu HL, Chuang CK, Jayakumar T, Wang CJ, et al. The role of protein-bound uremic toxins on peripheral artery disease and vascular access failure in patients on hemodialysis. Atherosclerosis. 2012. doi:10.1016/j.atherosclerosis.2012.07.012. 10.1016/j.atherosclerosis.2012.07.012 22981405

[45] Lin CJ, Wu V, Wu PC, Wu CJ. Meta-Analysis of the Associations of p-Cresyl Sulfate (PCS) and Indoxyl Sulfate (IS) with Cardiovascular Events and All-Cause Mortality in Patients with Chronic Renal Failure. PLoS One. 2015. doi:10.1371/journal.pone.0132589. 10.1371/journal.pone.0132589 PMC4501756 26173073

[46] Chen J, Hamm LL, Mohler ER, Hudaihed A, Arora R, Chen CS, et al. Interrelationship of Multiple Endothelial Dysfunction Biomarkers with Chronic Kidney Disease PLoS One. 2015. doi:10.1371/journal.pone.0132047. 10.1371/journal.pone.0132047 PMC4488859 26132137

[47] Cines DB, Pollak ES, Buck CA, Loscalzo J, Zimmerman GA, McEver RP (1998). Endothelial cells in physiology and in the pathology of vascular disorders. Blood.

[48] Dong J, Li YJ, Yang ZK, Xu R. Prognostic value of serum von Willebrand factor, but not soluble ICAM and VCAM, for mortality and cardiovascular events is independent of residual renal function in peritoneal dialysis patients. Perit Dial Int. 2014. doi:10.3747/pdi.2012.00004. 10.3747/pdi.2012.00004 PMC4269496 24584618

[49] Eugen-Olsen J, Andersen O, Linneberg A, Ladelund S, Hansen TW, Langkilde A, et al. Circulating soluble urokinase plasminogen activator receptor predicts cancer, cardiovascular disease, diabetes and mortality in the general population. J Intern Med. 2010. doi:10.1111/j.1365-2796.2010.02252.x. 10.1111/j.1365-2796.2010.02252.x 20561148

[50] Pawlak K, Myśliwiec M, Pawlak D. Kynurenine pathway - a new link between endothelial dysfunction and carotid atherosclerosis in chronic kidney disease patients. Adv Med Sci. 2010. doi:10.2478/v10039-010-0015-6. 10.2478/v10039-010-0015-6 20439183

[51] Stenvinkel P, Lindholm B, Heimbürger M, Heimbürger O. Elevated serum levels of soluble adhesion molecules predict death in pre-dialysis patients: association with malnutrition, inflammation, and cardiovascular disease. Nephrol Dial Transplant. 2010. doi:10.1093/ndt/15.10.1624. 10.1093/ndt/15.10.1624 11007832

[52] Avci E, Coskun S, Cakir E, Kurt Y, Ozgur Akgul E, Bilgi C. Relations between concentrations of asymmetric dimethylarginine and neopterin as potential risk factors for cardiovascular diseases in haemodialysis-treated patients. Ren Fail. 2008. doi:10.1080/08860220802249009. 10.1080/08860220802249009 18791952

